# Dancing with the patient: a qualitative study of general practitioners’ experiences of managing patients with multimorbidity and common mental health problems

**DOI:** 10.1186/s12875-023-02056-y

**Published:** 2023-04-20

**Authors:** C. Kappelin, C. Sandlund, J. Westman, C. Wachtler

**Affiliations:** 1 Department of Neurobiology, Care Sciences, and Society, Division of Family Medicine and Primary Care, Karolinska Institute, Alfred Nobel’s Allé 23, S-141 52, Huddinge, 141 52 Sweden; 2 Academic Primary Healthcare Centre, Solnavägen 1E, Stockholm, 113 65 Sweden; 3 Marie Cederschiöld University, Stigbergsgatan 30, Stockholm, 116 28 Sweden

**Keywords:** Multimorbidity, Common mental health problems, General practitioners, Social isolation, Treatment burden

## Abstract

**Background:**

Patients with multimorbidity, having two or more chronic diseases, suffer frequently from undiagnosed common mental health problems and are an increasing challenge in primary care. There is a call to improve care delivery to address all these patients’ needs at the same time. The aim of this study was to identify general practitioners’ experiences of managing patients with multimorbidity and common mental health problems in primary care.

**Methods:**

We conducted five focus group interviews with 28 physicians (3–8 participants in each group) in 5 primary care practices in and outside of Stockholm, Sweden. We used a semi-structured interview guide, and we analysed the data using reflexive thematic analysis. The methodological orientation of the study was inductive, latent constructivism.

**Results:**

We generated two themes from the data: *Unmet patient needs and fragmented care send patients and physicians off balance* and *Dancing with the patient individually and together with others leads to confident and satisfied patients and physicians.* The two themes are related as general practitioners expressed a need to shift from disease-specific fragmentation to relational continuity, teamwork, and flexibility to meet the needs of patients with multimorbidity and common mental health problems.

**Conclusions:**

These findings can provide guidance in developing future interventions for patients with multimorbidity and common mental health problems in primary care in general, and in Sweden in particular.

**Supplementary Information:**

The online version contains supplementary material available at 10.1186/s12875-023-02056-y.

## Introduction

Patients with multimorbidity, at risk of poorly detected common mental health problems (CMHPs) [1], are regular users of primary care. Multimorbidity is the co-occurrence of two or more chronic diseases [2] and increases as the population ages [3]. Patients with multimorbidity, with or without a previous CMHP, suffer more frequently than others from new CMHPs [4, 5]. In addition, patients with multimorbidity and CMHPs suffer from poor quality of life [6, 7]; increased mortality and health care costs [7]; and poorer mental [8] and physical [9] health outcomes then if treating each condition alone [10]. However, CMHPs are often undetected and/or undiagnosed in this patient group [11] .

Patients with multimorbidity and CMHPs need improved care delivery. The World Health Organisation (WHO) [2], The National Institute for Health and Care Excellence (NICE) [12], and The Academy for Medical Sciences [13] have highlighted the need of a more integrated care delivery approach to address all these patients’ diseases and needs at the same time. Improvement of care for individuals with multimorbidity and CMHPs has been prioritized in Sweden, and Region Stockholm recently highlighted the need for improved patient continuity with both a general practitioner (GP) and a district nurse for this group [14].

The health care system in Sweden is organised with primary care practices having a gatekeeper function to secondary care, with the ambition of full empanelment. However, only a third of the population have a named GP [15, 16]. The funding of the Swedish health care system is primarily regional. However, private actors have increased over the past decades, yet having structures for cooperation with the Regions [17, 18]. Swedish GPs see fewer patients but suffer from more stress than colleagues in other western countries, potentially because of poor continuity. In addition, Swedish primary care has poor structures to interact with other healthcare providers and meet patients with chronic illnesses compared with primary care in other western countries [19]. In Region Stockholm, the number of privately financed both primary and secondary care practices are the highest in the country. The population living in Region Stockholm are the highest consumers of primary care, despite being relatively healthy and younger compared to the rest of the country [20].

Collaborative care (CC) is a complex intervention that has shown positive results for patients with multimorbidity involving depression, yet further research is needed [21–23]. CC is a care model for teamwork between the patient, the GP and a nurse care manager. The care manager and the patient set up a structured care plan, often involving medication or psychotherapy, and have scheduled patient follow-ups. Moreover, the GP and the nurse care manager have a structure for inter professional communication [24]. CC has shown positive results in primary care settings in reducing depressive symptoms in patients with depression both internationally [25] and in Sweden [26], in reducing anxiety symptoms in patients with anxiety [25], as well as in reducing depressive symptoms in patients with multimorbidity involving depression and one more chronic disease [22]. However, there are no studies showing CC to be effective for patients with multimorbidity involving anxiety [23], for patient with complex multimorbidity [22, 23], or set in a Swedish context [22]. Optimal development, testing, evaluation and implementation of complex interventions like CC in a new context requires attention and adaptation [27].

Relatively little is known about the perspectives of GPs who daily manage patients with multimorbidity and CMHPs. A recent systematic review examined GPs’ experiences of managing patients with multimorbidity included 33 qualitative studies set in 14 countries in Europe, North America, and Australia [28]. However, only one study, set in Australia, examined GPs’ views on multimorbidity with CMHPs. That study reported views of 8 physicians about detecting depression in patients with multimorbidity [29]. Understanding how GPs working in Sweden experience care delivery and their thoughts on how to improve it, is critical in order to contextualize development of a CC-based complex intervention to improve care delivery for patients with multimorbidity involving CMHPs in Sweden [27, 30].

The aim of this study was to identify GPs’ experiences of managing patients with multimorbidity and CMHPs in primary care in Stockholm, Sweden.

## Methods

This qualitative study is a cross-case reflexive thematic analysis of focus group (FG) interviews [31]. We chose reflexive thematic analysis, developed by Braun and Clarke [31, 32], as this approach enabled us to be flexible and thoroughly analyse the data when forming codes and developing themes from the data. The underlying theoretical framework and methodological orientation of the study was inductive, latent constructivism. This means our approach was interpretative and aimed at creating an understanding of the multiple experiences of the group we interviewed [33].

We followed the COREQ guidelines [34] and a checklist for reporting reflexive thematic analysis [32] for reporting this qualitative study.

### Researcher backgrounds

CK is a resident physician in primary care and a PhD-student. She conducted the five FG interviews. CW is a GP, PhD in Family Medicine, and an experienced qualitative researcher. She participated and observed in one of the FG interviews to follow up questions of interest in the focus group and to provide feedback on interviewing technique to CK after the interview. CS and JW, being district nurses, PhD and professor respectively, participated in the data analysis. Both had previous experiences of qualitative analysis. All authors were part of the same research group aiming to develop a primary care intervention to improve care delivery for patients with multimorbidity and CMHPs. One more district nurse, initially being part of the same research group but dropping out before the analytical process began, observed two interviews, adding questions to the focus groups when needed. All researchers were female.

### Participant selection

We used purposive sampling to identify practices with private or public financing, different sizes and from different areas in and outside of Stockholm (Table 1). CK and CW identified five practices at which they had existing contacts. CK approached the heads of the practices by email to ask if there were physicians willing to participate in an FG interview. All practices accepted. The research team offered the participants lunch during the interviews. A total of 28 participants were included in the study (Table 2). Three to eight participants were present in each group. No participating practice nor individual participant refused to participate or dropped out.

CK knew one participant in two FGs collegially but had no prior relationship with other participants. CW did not have any relationship with the participants in the interview that she participated in. However, CW worked in one of the practices where interviews were conducted but did not participate in that interview. Participants had information about the interviewer’s professional background and the aim of the research group to improve care delivery for patients with multimorbidity and CMHPs.

To estimate sample size needed to answer the research question, CK and CW discussed information power [35]. Information power is related to five aspects of study design and high information power: *Study aim*, where information power increases with a narrow and decreases with a broader aim; *Specificity*, regarding the participants knowledge in the field to be dense increasing or sparse decreasing information power; using an *Established theory* to analyse the data, increases information power; *Quality of dialogue*, where strong quality increases and weak quality decreases information power; and analysis being one case, increasing or cross-case decreasing information power [35]. In this study we have a dense sample specificity, as participants have years of experience and deep knowledge of the topic, and a good quality of dialogue, because of the semi-structured interview form, increasing information power. However, as we intended to explore the participants experiences constructively and inductively, we used a broad study aim and did not use an established theory to analyse the data. Moreover, we used an exploratory cross-case analysis further decreasing information power. Based on these conditions and previous FG research experience [35], we considered adequate information power could be achieved with 20–30 participants in 4–5 FGs.

**Table 1:** Information about the participating primary care practices

Primary care practice	Number of listed patients	Financing of the practice (Public or private)	Location (Suburban or Urban)
P1	10.000-19.999	Public	Suburban
P2	≥ 30.000	Public	Urban
P3	20.000-29.999	Private	Suburban
P4	20.000-29.999	Public	Suburban
P5	< 10.000	Private	Suburban

**Table 2:** Information about the 28 participating physicians in the focus-group interviews

Profession	Age (in years)	Sex
15 General Practitioners 1 Geriatrician 4 Resident physicians in in General Practice 4 Medical interns 4 Assistant physicians	26–67	19 women 9 men.

### Data collection

The research group developed a semi-structured interview guide (Additional file 1). It included questions about:

- Managing patients with multimorbidity and CMHPs.
- How to improve care delivery for these patients.
- Participant reflections on the CC Model for these patients.

CK pilot-tested the interview guide in an FG with five physicians in a separate PC practice prior to the first FG interview and made minor revisions to the interview guide. Three FG interviews were conducted face-to face in the practices. Due to the on-going Covid-19 pandemic, the remaining FGs were conducted on Zoom with participants sitting in separate rooms with camera and audio on. Only participants and the researchers were present. Interviews took place November 2020 until January 2021, lasted 40 to 60 min, were audio recorded, and were transcribed by a transcription company. CK took field notes after each interview. No repeated interviews were carried out. Transcripts were not returned to the participants for comments.

### Data analysis

All authors read the transcripts and documented ideas of interest independently. CK coded all data. Codes were short phrases, data-driven, and predominantly semantic, that identified meaningful units of data for the research topic from the whole data set. CW coded data from one interview and the two sets of codes for that interview were used for feedback and mentorship on the coding process. CK read all codes and reread transcripts, ensuring all relevant information was coded. CK and CW reviewed all codes together and CW gave feedback on the coding matrix.

CK categorized codes into descriptive subthemes. Together, all authors ensured the descriptive subthemes reflected the data, implementing ideas of interest from reading the manuscript independently from before, and discussed and revised the subthemes into more analytical ones iteratively. Authors developed two analytical themes based on discussions about how the subthemes were related. CK checked that the analytical themes covered relevant data by reciprocal reading of the entire data set. Authors rearranged and refined the themes. CK read the transcripts again to check that the themes were representative. CK and CW wrote up a definition of each theme and subtheme and described how subthemes related to each other. CK wrote the first draft of the report, identifying illustrative citations and further refining theme and subtheme names. All authors contributed to further iterations of the report. Participants were not asked to provide feedback on the findings.

Data were managed in excel and PowerPoint during the analytic process. The interviews and data analysis were conducted in Swedish. CK translated themes and subthemes and translation was checked by CW, a native English speaker.

## Results

We generated two themes, involving two and three subthemes respectively, in our analysis:

*Unmet patient needs and fragmented care send patients and physicians off balance* and *Dancing with the patient individually and together with others leads to confident and satisfied patients and physicians.*

The two themes are related, showing that balanced, individualized patient care requires a shift from disease-specific fragmentation to relational continuity, teamwork, and flexibility.

### Unmet patient needs and fragmented care send patients and physicians off balance

For physicians, patients with multimorbidity and CMHPs needed a holistic approach not provided by the current fragmented health care system. This imbalance led to patient treatment burden and ethically stressed and frustrated physicians. They expressed that Isolation and unmet patient needs cause poor patient mental health. Moreover, they described failing to address poor patient mental health because Fragmented care burdens patients and frustrates physicians.

#### Isolation and unmet patient needs cause poor patient mental health

Physicians understood poor mental health in patients with multimorbidity due to poorly addressed needs and patient isolation, rather than a specific mental illness.

*“We believe it’s the isolation at home that is… the primary trigger of depression” R3, P3*.

Isolation and unmet needs, rather than the patients’ exact number of diseases, were seen as drivers of patient wellbeing and quality of life. This included: social aspects, including loneliness and poor social context; physical aspects, including symptom burden, pain, and physical impairment; mental aspects, including psychiatric diseases, alcohol abuse, and increased late-life existential thoughts; and poor self-efficacy, because of frailty, cognitive impairment, old age, economic status, poor language knowledge, and poor digital knowledge.

*“I think their depression is often multifactorial. It can often involve pain, loneliness, maybe too much alcohol. Worries about life and death – existential thoughts increase when you are severely ill” R1, P2*.

Isolation and unmet needs did not fit into current health care priorities and physicians found it difficult to address them in their daily work. They lacked clear management structures for multimorbidity and CMHPs in older patients. New symptoms were often interpreted as having a somatic cause in this patient group, rather than connected to untreated CMHPs. Furthermore, they found patients with CMHPs responded poorly to antidepressant medication, had poor access to psychologists, and lacked access to social workers.

*“Sometimes it feels like mental ill-health in older and multimorbid patients is a bit forgotten actually. Maybe it is me that is bad at picking up on it. But sometimes, having a lot of somatic conditions to treat, it can sometimes in a way fall through the cracks. How they actually feel. It is sometimes easy to, in a way, bulldoze it aside thinking, well they have so many medications and other issues…” R1, P4*.

Although isolation was considered a cause of mental health problems in patients, it was not seen as a primarily medical responsibility. Some physicians suggested that the unit for older patients present in some practices should be responsible, while others suggested the municipal services should be responsible. However, physicians felt their knowledge of, access to, confidence in, and cooperation with the unit for older patients and the municipal services to vary and sometimes fail.

*“I believe, with respect to how prevalent and common it is, the municipal services should provide some kind of contact with a social worker. Because it is not reasonable that it falls back at the primary care practice, that a person is lonely.” R5, P1*.

#### Fragmented care burdens patients and frustrates physicians

The current health care system focuses on single diseases causing physicians ethical stress due to increased risk of patient treatment burden and frustration when they could not meet patients’ needs and expectations.

*“Because the patient doesn’t fit. Disease specific guidelines by all means, but I am a bit allergic to them.” R1, P1*.

For patients with multimorbidity and CMHPs, physicians described how the system led to many short visits for each of the patients’ diseases and new symptoms. This led to contacts with different physicians and subspecialist nurses to prescribe or follow-up new medications and new recommendations for the patients with poor inter-professional communication both in and outside of the primary care unit for the physicians.

*“I actually have several patients where we discuss having many health care contacts takes a lot of energy.” R3, P5*.

The physicians felt this silo-mentality risked burdening the patient with polypharmacy with adverse effects, risk of addiction, and medication interactions. It also led to more health care contacts, recommendations, and prescriptions than the patient can manage, leading to poor compliance and poor health results.

*“…I can imagine that … we skimp on a lot with the diagnostics and that a lot of symptom-relieving medications are prescribed perfunctorily. A lot of patients are prescribed benzodiazepines, a lot of patients are prescribed Zopiclone regularly. Due to anxiety without further specification…” R2, P4*.

GPs expressed frustration trying to help patients, squeezing in patients with multimorbidity and CMHPs into rigid structures of narrow disease-specific guidelines. They described unsatisfied, worried patients booking frequent unscheduled appointments showing no effect of given care.

*“If you see it from the patient’s perspective, bringing their long lists to their annual visit, it is indeed different agendas where we know that there is really a lot we need to follow up, do and decide (…). You really try to conjure at those annual visits, yet that is not so good either.” R3, P5*.

They were frustrated by having to evaluate possibly new symptoms in a patient they do not know, with many diseases and medications to consider, in brief visits without the possibility for follow-up appointments.

Seeing a new patient with multimorbidity and psychiatric issues. It really takes time to sort out what is what. And what kind of help the patient needs. Considering the lack of available appointments in the health care unit, it can really drag on.” R5, P2

GPs expressed ethical stress due to care-access inequities in this group. They described some older isolated patients quietly put up with their symptoms while patients with high self-efficacy had good access. Access was especially difficult for patients who could not use digital services or had poor language skills. In addition, physicians had little resources to conduct home visits.

*“Patients taking a great responsibility for their own health and with the urge to seek help, I believe they often get good help. But I believe we have, we do have a worse result for more quiet patients not contacting the practice by themselves. It is easy that they are, well, forgotten. (…)” R3, P4.*

*Dancing with the patient individually and together with others leads to confident and satisfied patients and physicians.*

Physicians described using three interrelated strategies involving to balance care with the individual needs of their patients to be able to dance with their patients with multimorbidity and CMHPs. Following your patient involved to follow the patient over time to get to know one another and create safe long-lasting relationships. Taking turns with your patient meant having the mandate and time to adapt care to be able to respond to the patients’ individual needs. Interacting with and around your patient involved working together with the patient, and other healthcare providers such as the district nurse, and the municipal services, to provide care to meet the patients’ individual needs. Active use of these strategies together led to confidence and satisfaction enabling dancing for all parts.

#### Following your patient

Physicians pointed out the value of knowing your patient by following the patient over time, building relationships and trust. Knowing your patient was a key factor for safe and confident patients. GPs felt continuity could reduce unnecessary worry and un-scheduled contacts with the primary care practice. Following your patient was rewarding and made the physicians’ work easier by helping to see new symptoms, change over time, and for decisions about medication withdrawal.

*“A continuous contact with a physician. Definitely. And preferably a nurse. And to feel. They (the patients) should know who to contact when. And know that someone knows me. It is always much easier to help someone that you know. Someone that you understand. Because we… we dance with our patients. Or at least I do anyway. And then I need to know if it is tango or if it is waltz or salsa to dance. That I learn when I meet my patients.” R2, P1*.

#### Taking turns with your patient

Physicians identified a need for individualised care rather than following disease-specific guidelines or finding further quick fixes and new medications. This meant understanding and addressing the cause of the patients’ problems, including loneliness, existential thoughts, and behavioural and physical activity, as well as sitting back and listening, not always trying to solve an unsolvable problem.

*“(…) Sometimes we just have to listen. We just need to be there. To absorb it like a sponge. But sometimes we can have performance requirements and expectations on ourselves to do things and then we don’t want to hear. ‘Don’t tell me that, because I can’t do anything about it.’ But to nevertheless dare to be a bit brave and say ’I listen. I can’t do anything about it, but I can listen’ (…)” R5, P1.*

Physicians wanted flexibility to address the patients’ agenda and to structure the visits based on patient needs. Physicians also wanted room to do their doctor work: to investigate and diagnose symptoms, to go through the patients’ medical record and medications thoroughly, and to ask about and diagnose CMHPs more accurately.

*“I believe, in a perfect world, that you should think in a perfect world of the health care system. Then you would have a certain number of patients in your own patient list. And then I want that it is me they see for all their problems. And I would like to have enough time in their annual visits to go through everything. To go through their damn long lists so that they do not need to come 15 times, because that makes it fragmented. And to integrate mental health. Because that is really something.” R1, P5.*

Physicians felt scheduled patient follow-ups to address new symptoms and mental ill-health, as well as improved access, could lead to confident patients and fewer unscheduled contacts. Some already used informal structures for scheduled patient follow-ups to support patients with multimorbidity and worry who had frequent contact with the primary care practice. Physicians also wanted to increase accessibility for patients to book appointments themselves, including using a calling system to avoid missing patients. Moreover, they wanted more time in their schedule to make follow-ups visits at home.

*“And preferably, a lot more time to book follow-ups visits. Both planned visits and accessibility for the patients to contact the practice if they have something on their minds. I believe, it creates confidence.” R3, P4*.

#### Interacting with and around your patient

To improve physician and patient confidence, physicians expressed the importance of sharing patient relational continuity and responsibility with other professionals to address aspects of isolation.

*“In a perfect world, we would work more in teams. That you actually have someone to discuss your difficult patients with differently from what we do today.”* R2, P2.

They proposed teamwork with the district nurse could address social situation and behavioural activation, be one more pair of eyes on the patient, and to follow up medication adherence. Physicians proposed sharing a patient list and care plan with nurses to improve interprofessional communication in the primary care practice. They suggested scheduled meetings to discuss the patients. Physicians suggested that patients could see several health care providers at the primary care practice at one visit to work more holistically and reduce number of visits. Physicians drew parallels to positive experiences of existing structures of teamwork in the primary care units such as home health care for the sickest older patients with multimorbidity, where physicians work in teams with a nurse with a clear structure of communication simplifying work, enabling relational continuity and room to individualise care giving more confident patients.

*“I am thinking a care plan. That you involve a nurse in the primary care practice and work together with. Because it often works well in home health care when they finally end up there with me. Then they meet the nurse every other week and get five minutes to just talk. And that is quite enough. And then I get reports now and then if something happens and visit the patients once every six months. To collaborate together. . I am thinking about the ones contacting the primary care practice frequently, to have a plan when they call, what you say and how you should think. That you collaborate physician and nurse around them.”* R1, P1.

Furthermore, in some primary care practices, physicians described having developed their own structures of care, using a nurse to help older patients for medication follow-ups and counselling, to better address patients with multimorbidity, with positive results.

*“But if we do recognise them, they are quite well taken cared of. On the one hand, we have psychologists. And then, we have a nurse who should address this type of patients. When you prescribe a new medication, she can do follow-ups. And she can have follow-ups by telephone about wellbeing and that sort of things. So, that actually works quite well here.”* R1, P3.

Some physicians suggested broadening knowledge in the practice to better address patients with multimorbidity and CMHPs, for example by using social workers who could address social context and existential thoughts, or pharmacists to help with medical reviews to reduce polypharmacy.

*“I was thinking about a general need of a pharmacist, someone who can go through the patient’s medications more thoroughly than we can.”* R3, P2.

Physicians described needing improved interprofessional communication outside of the practice. They suggested primary care nurses could liaise between patients and the municipality to better address patients’ social needs. Physicians also wanted better cooperation with other health care providers such as geriatrics clinics and the geriatric psychiatric clinics. Finally, physicians pointed out that better collaboration between primary care, secondary care and municipality services could lead to improved care.

*“We need cooperation, it is a lot with municipality and the health care system (…)” R1,P1*.

## Discussion

In our analysis of FG interviews with GPs about managing patients with multimorbidity and CMHPs, we generated two themes:

*Unmet patient needs and fragmented care send patients and physicians off balance* and *Dancing with the patient individually and together with others leads to confident and satisfied patients and physicians.*

Patient isolation and poorly addressed needs were seen as a cause of mental health problems in patients with multimorbidity, with inequalities in quality of and access to care resulting in ethically stressed physicians. Patients often get too much of what they do not need, including medications, short-sighted solutions, and multiple provider visits, leading to burdened patients and frustrated physicians. GPs described working to pivot from a fragmented approach to provide individualized, flexible care based on relational continuity with one physician, using teamwork between the patient, the physician, a primary care nurse, and other health care providers in and outside of the primary care practice as well as the municipal services. Dancing with the patient like this was seen to promote confident and satisfied patients and physicians.

Our findings are in line with the existing literature about GP experiences managing patients with multimorbidity [28], but our study adds some important perspectives about management of patients with multimorbidity and CMHPs. Our findings are in line with the systematic literature review regarding GPs experiences of patients with multimorbidity, identifying risk of treatment burden for patients with multimorbidity in today’s fragmented health care system, and identified relational continuity and patient centred care as strategies for managing these patients [28]. In addition, frailty, high age, social, cultural, and economic factors, and poor self-efficacy and ability were understood to lead to more complex multimorbidity, as in our study. Beyond these similarities, GPs in our study experienced CMHPs as stemming from patient isolation and unmet patient needs regarding physical and mental symptoms, social support, and self-efficacy. Additionally, GPs identified that imbalance between what patients need and what they receive leads to ethically stressed and frustrated physicians. The single previous study of physicians’ experiences identifying and diagnosing depression among patients with multimorbidity [29] identified relational continuity as facilitating diagnostics and identified both medical and social interventions as necessary to meet these patients‘ needs. In our study we also highlight the need of teamwork both in and outside of the primary care practice to properly help patients with multimorbidity and CMHPs. Beyond teamwork and relational continuity, GPs in our study also stressed the importance of actively working to make room for the patient’s individual needs.

The theme *Unmet patient needs and fragmented care send patients and physicians off balance* is in line with the Cumulative Complexity Model for multimorbidity (CCM) [36, 37]. CCM describes how imbalance between reduced capacity due to physical and social factors, combined with increased workload due to an increased number of health care contacts and medications, lead to an increased burden of illness and treatment for patients with multimorbidity. GPs in our study described an imbalance between unmet patient needs and care provided by the current health care system. They saw this imbalance as a cause of patient mental health symptoms. This study adds the critical aspect of how this imbalance not only negatively affects patients, but also GPs.

In our study, GPs pointed out that addressing isolation and unmet patient needs were vital to improve mental health in patients with multimorbidity, and that these factors were more important than the number of diagnoses. Recent studies use the term ‘complex multimorbidity,’ commonly defined as having three or more chronic diseases [38], to identify severely ill patients [2]. This definition may be more useful for identifying patients in need of interventions. However, the term does not address social factors [39]. The importance of physical symptoms [40] and social isolation [41] in developing CMHPs are known. This suggests addressing social factors in future primary care intervention development may be meaningful in improving mental health in patients with multimorbidity. Regarding the use of CC in complex intervention development for these patients, the care model involves teamwork between a GP and a district nurse, and a structure for relational continuity which the GPs ask for. However, the structured management plan fails to correspond to patients with multimorbidity and CMHPs’ needs of individualised care and flexibility to address aspects of physical, mental, and social needs [21–23]. Moreover, CC often lacks structures for teamwork outside of the primary care practice, such as with the municipalities. The structure of CC can be applied in a future complex intervention design, but it must be refined regarding the content of the structured management plan and the involvement of teamwork outside of the primary care practice according to the participating GPs in this study.

Finally, our findings point out that GPs experience frustration and ethical stress when they are limited to disease-specific fragmented care for this patient group. WHO [2] and the Academy of Medical Sciences [13] have highlighted the need of a changed priority to address the aging population with increased risk of both multimorbidity and isolation. WHO is currently leading the Decade of a Healthy Aging to support community and health-care system development to meet older patients’ needs [2]. This is what the GPs in our study ask for.

This study has some methodological strengths and weaknesses. We used the concept of information power [35] to guide sample size. This concept is commonly used in our chosen methodology [31, 32]. Moreover, as we aimed to identify patterns in the data rather than to identify all possible aspects of GP experiences, we found information power to be the appropriate concept to use in this study. Purposive sampling allowed us broad access to GPs’ experiences and thoughts, improving the specificity aspect of information power. Participants had varying lengths of experience working in primary care, enabling us to have a broader perspective of both young and more experienced GPs’ points of view, as well as physicians’ experiences from both in and outside of Stockholm working in different socioeconomic areas. There could be opinions or thoughts in further focus groups that would have deepened our analytical process, in Region Stockholm or other parts of Sweden. However, our results reflect a phenomenon in GPs in and outside of Stockholm and our results are congruent with other international qualitative studies, without the perspective of CMHPs, in similar settings [28]. Participants were colleagues, which may have promoted recruitment and participant trust in the researchers. CK knew two participants collegially which might have affected what participants said about the topic. Reflexive thematic analysis allowed us to be flexible and thoroughly analyse the data when inductively forming latent codes and constructing themes from the data. CK and CW are physicians in general practice and had therefore preunderstandings that could lead to assumptions of what participants meant during the interviews and during analysis. Involvement of CS and JW, both district nurses, in the analytical process widened the preunderstanding in the group. However, all researchers are health care workers with deep knowledge of primary care and an interest in improving primary care delivery, thus likely to focus on problems in primary care. Hence, we risked missing an outsider perspective. These risks of bias were identified prior to data collection and the group agreed to take a step back and be open-minded during the interviews and in the analytical process avoiding assumptions. We also actively used our pre-understanding in the analytical process to identify patterns in the data. CK was a PhD-student without former experience in FG interviewing and risked not following up questions enough to capture what the participants really meant, risking premature closures that could affect data quality. For this reason, CW audited an FG interview to give supervision and feed-back, CK had no prior experience in thematic analysis when she generated the initial codes, so the coding process was supervised by CW. All authors supported iterative checking of themes, codes, and data.

## Conclusion

This study indicates that GPs see CMHPs in patients with multimorbidity as resulting from isolation and unmet patient needs, poorly addressed by a fragmented structure of care designed for patients with single conditions, acute symptoms, and high self-efficacy. GPs feel frustrated and ethically stressed by the situation, but they work to provide patient-centred care as needed using relational continuity and teamwork with the patient and other health care providers. GPs want clarified responsibility for addressing isolation and other unmet patient needs, especially social ones. They want to improve inter-professional communication within and outside of the PC unit, as well as with the municipality. These findings can be used by clinicians and policymakers to prioritise and develop primary care delivery for patients with multimorbidity and CMHPs in general and in the context of Region Stockholm, Sweden in particular. This study will be used as a starting point for development of a complex intervention for this patient group in Region Stockholm, Sweden.

## Electronic supplementary material

Below is the link to the electronic supplementary material.

**Supplementary Material 1**

**Supplementary Material 2**

## Data Availability

An example of the data and data analysis supporting the findings of this article is present in the supplementary information files (Additional file 2). The full datasets used and analysed during the current study are available from the corresponding author on reasonable request.

## List of abbreviations

CMHP: Common mental health problem
GP: General practitioner
CC: Collaborative care
FG: Focus group
