# Supplementary material for: Dancing with the patient: a qualitative study of general practitioners’ experiences of managing patients with multimorbidity and common mental health problems
Source: BMC Prim Care. 2023 Apr 20;24:104. doi: 10.1186/s12875-023-02056-y (PMC10117273; doi:10.1186/s12875-023-02056-y)
Supplement: Supplementary file 1 — Supplementary Material 1 [file 12875_2023_2056_MOESM1_ESM.docx]

**Additional file 1.**

**Semi structured interview guide**

- Presentation of the interviewer and the study aim. Information that the participants can stop their participation at any time point during the interview.

1. Do you have any questions before we start this interview?
2. Then we start the interview. Would you like to present yourselves with name, age, working title, and working place?
3. Would you like to describe a case or a typical case of a patient with multimorbidity for you?
4. How do the connection to common mental disorders, such as depression and anxiety, look for the patients with multimorbidity?
5. Can you give examples of how care is delivered for patients with multimorbidity and common mental disorders in your primary care unit?
   1. What works?
   2. What is difficult?
   3. What do the patients need?
   4. What do you need?
   5. How is care provided for a patient with two chronic diseases such as cardiovascular diseases and a potential diagnosis of depression or anxiety?
6. How would you like the care to be delivered for these patients in a perfect world?

- I will now present a care model for you called collaborative care, which are effective in reducing depressive symptoms in patients with multimorbidity and depression and have been tried out in a Swedish primary context with positive results for patients with depression alone.
  1. A nurse in the primary care unit will have training in collaborative care before the intervention starts.
  2. The patient sees the nurse – a care manager - in the primary care unit and set up a care plan involving behavioural activation, and information on the patients’ diseases.
  3. Furthermore, they have scheduled patient follow-ups where they can discuss the patient’s wellbeing, level of functioning and compliance
  4. The nurse has regular contact with you reporting on the patient’s wellbeing and if need of changes of medications.
  5. Moreover, the nurse will have regular supervision with a psychologist or a specifically interested physician in the primary care unit.

1. Have anyone of your heard of or experience from working in this model from before?
   1. Can you elaborate?
2. Regarding to use this care model in Swedish primary care, we would like to know your thoughts. Can you please tell us?
   1. What could be positive or negative with this type of model?
   2. Possibilities and difficulties to implement?
3. In what way would this way of working help you in your daily work?
4. Other thoughts or reflections? Anything that you would like to add?

- Thank you for participating in this interview.
