# Supplementary material for: Dancing with the patient: a qualitative study of general practitioners’ experiences of managing patients with multimorbidity and common mental health problems
Source: BMC Prim Care. 2023 Apr 20;24:104. doi: 10.1186/s12875-023-02056-y (PMC10117273; doi:10.1186/s12875-023-02056-y)
Supplement: Supplementary file 2 — Supplementary Material 2 [file 12875_2023_2056_MOESM2_ESM.docx]

**Additional file 2.**

**An overview of the included themes and subthemes and examples of codes and quotes for each of the themes.**

| **Theme** | **Subtheme** | **Code** | **Quote** |
| --- | --- | --- | --- |
| **Unmet patient needs and fragmented care send patients and physicians off balance** | *Isolation and unmet patient needs cause poor mental health* | Isolation= most important trigger for mental health problems | *“We believe it’s the isolation at home that is... the primary trigger of depression” R3, P3* |
|  |  | MM=Mental ill-health is often multifactorial | *“I think their depression is often multifactorial. It can often involve pain, loneliness, maybe too much alcohol. Worries about life and death – existential thoughts increase when you are severally ill” R1, P2* |
|  |  | MM= mental ill-health is often forgotten | *“Sometimes it feels like the mental illness in elderly and multimorbid patients is a bit forgotten actually. Maybe it is me that is bad at picking up on it. But sometimes, having a lot of somatic conditions to treat, it can sometimes in a way fall through the cracks. How they actually feel. It is sometimes easy to, in a way, bulldoze it aside thinking, well they have so many medications and other issues(...)” R1, P4* |
|  |  | Loneliness= leads to common mental health problems | *“I believe, with respect to how prevalent and common it is, the municipal services should provide some kind of contact with a social worker. Because it is not reasonable that it falls back at the primary care practice, that a person is lonely.” R5 ,P1* |
|  | *Fragmented care burdens patients and frustrates physicians* | The patients don’t fit | *“Because the patient doesn’t fit. Disease specific guidelines by all means, but I am a bit allergic to them.” R1, P1* |
|  |  | Many visits take a lot of energy | *“I actually have several patients where we discuss having many health care contacts takes a lot of energy.” R3, P5* |
|  |  | Symptom revealing medications= Quick fix to a complex problem | *“…I can imagine that it… skimps on a lot with the diagnostics and that a lot of symptom-relieving medications are prescribed perfunctorily. A lot of patients are prescribed benzodiazepines, a lot of patients are prescribed Zopiclone regularly. Due to anxiety without further specification…” R2, P4* |
|  |  | Patient and physician agenda differs | *“If you see it from the patient’s perspective, bringing their long lists to their annual visit, it is indeed different agendas where we know that there is really a lot we need to follow up, do and decide (…). You really try to conjure at those annual visits, yet that is not so good either” R3, P5* |
|  |  | It takes time | *“Seeing a new patient with multimorbidity and psychiatric issues. It really takes time to sort out what is what. And what kind of help the patient needs. Considering the lack of available appointments in the health care unit, it can really drag on.” R5, P2* |
|  |  | Quit patients are forgotten | *“Patients taking a great responsibility for their own health and with the urge to seek help, I believe they often get good help. But I believe we have, we do have a worse result for more quit patients not contacting the practice by themselves. It is easy that they are, well, forgotten. (…)” R3, P4* |
| **Dancing with the patient individually and together with others leads to confidant and satisfied patients and physicians** | *Knowing your patient* | Relational continuity = gets safe patients | *“A continuous contact with a physician. Definitely. And preferably a nurse. And to feel.. They (the patients) should know who to contact when. And know that someone knows me. It is always much easier to help someone that you know. Someone that you understand. Because we… we dance with our patients. Or at least I do anyway. And then I need to know if it is tango or if it is waltz or salsa to dance. That I learn when I meet my patients.” R2, P1* |
|  | *Making room for the patient* | Dare to just listen | *“(…) Sometimes we just have to listen. We just need to be there. To absorb it like a sponge. But sometimes we can have performance requirements and expectations on ourselves to do things and then we don’t want to hear. ‘Don’t tell me that, because I can’t do anything about it.’ But to nevertheless dare to be a bit brave and say ’I listen. I can’t do anything about it, but I can listen’ (…)” R5, P1.* |
|  |  | Individualised care to provide care as needed | *“I believe, in a perfect world, that you should think in a perfect world of the health care system. Then you would have a certain number of patients in your own patient list. And then I want that it is me they see for all of their problems. And I would like to have enough time in their annual visits to go through everything. To go through their damn long lists so that they do not need to come 15 times, because that makes it fragmented. And to integrate mental health. Because that is really something.” R1, P5.* |
|  |  | Scheduled follow-up visits= more confident patients | *“And preferably, a lot more time to book follow-ups visits. Both planned visits and accessibility for the patients to contact the unit if they have something on their minds. I believe, it creates confidence.” R3, P4* |
|  | *Working together with the patient* | A need to discuss difficult patients | *“In a perfect world, we would work more in teams. That you actually have someone to discuss your difficult patients with differently from what we do today.”* R2, P2. |
|  |  | Collaboration between physician and nurse – works well in home health care | *“I am thinking a care plan. That you involve a nurse in the primary care practice and work together with.. Because it often works well in home health care when they finally end up there with me. Then they meet the nurse every other week and get five minutes to just talk. And that is quite enough. And then I get reports now and then if something happens and visit the patients once every six months. To collaborate together . . . I am thinking about the ones contacting the primary care practice frequently, to have a plan when they call, what you say and how you should think. That you collaborate physician and nurse around them.”* R1, P1 |
|  |  | Existing well-functioning follow-ups by a nurse | *“But if we do recognise them, they are quite well taken cared of. On the one hand, we have psychologists. And then, we have a nurse who should address this type of patients. When you prescribe a new medication, she can do follow-ups. And she can have follow-ups by telephone about wellbeing and that sort of things. So, that actually works quite well here.”* R1, P3 |
|  |  | A pharmacist for medical reviews | *“I was thinking about a general need of a pharmacist, someone who ca go through the patient’s medications more thoroughly than we can.”* R3, P2. |
|  |  | We need improved cooperation outside of the primary care practice | *“We need cooperation, it is a lot with municipality and the health care system (…)” R1, P1* |
